# The impact of microRNA-mediated PI3K/AKT signaling on epithelial-mesenchymal transition and cancer stemness in endometrial cancer

**DOI:** 10.1186/s12967-014-0231-0

**Published:** 2014-08-21

**Authors:** Peixin Dong, Yosuke Konno, Hidemichi Watari, Masayoshi Hosaka, Masayuki Noguchi, Noriaki Sakuragi

**Affiliations:** Department of Women’s Health Educational System, Hokkaido University School of Medicine, Hokkaido University, N15, W7, Sapporo, 0608638 Japan; Department of Gynecology, Hokkaido University School of Medicine, Hokkaido University, N15, W7, Sapporo, 0608638 Japan; Division of Cancer Biology Institute for Genetic Medicine, Hokkaido University, N15, W7, Sapporo, 0608638 Japan

**Keywords:** Microrna, PI3K, PTEN, AKT, mTOR, EMT, Invasion, Cancer stem cell, Chemoresistance, Endometrial cancer

## Abstract

Activation of the PI3K/AKT pathway, a common mechanism in all subtypes of endometrial cancers (endometrioid and non-endometrioid tumors), has important roles in contributing to epithelial-mesenchymal transition (EMT) and cancer stem cell (CSC) features. MicroRNAs (miRNAs) are small non-coding RNA molecules that concurrently affect multiple target genes, and regulate a wide range of genes involved in modulating EMT and CSC properties. Here we overview the recent advances revealing the impact of miRNAs on EMT and CSC phenotypes in tumors including endometrial cancer via regulating PI3K/AKT pathway. MiRNAs are crucial mediators of EMT and CSC through targeting PTEN-PI3K-AKT-mTOR axis. In endometrial cancer cells, miRNAs can activate or attenuate EMT and CSC by targeting *PTEN* and other EMT-associated genes, such as *Twist1*, *ZEB1* and *BMI-1*. More detailed studies of miRNAs will deepen our understanding of the molecular basis underlying PI3K/AKT-induced endometrial cancer initiation and progression. Targeting key signaling components of PI3K/AKT pathway by restoring or inhibiting miRNA function holds promise as a potential therapeutic approach to suppress EMT and CSC in endometrial cancer.

## Introduction

Endometrial cancer (EC) is the most common invasive neoplasm of the female genital tract in the United States and many other developed countries. In 2014, there are about 52630 new cases and 8590 deaths due to this neoplasm [1]. Although Asian women have a lower risk of EC compared to those in the US and other western countries, the incidence of EC in Shanghai and in Japan has substantially increased [2,3].

A dualistic model of EC has been proposed, broadly classified into type 1 (approximately 75% of cases, endometrioid EC) and type 2 non-endometrioid tumors (serous and clear-cell histology) [4]. Most type 1 ECs are usually diagnosed early and have a good prognosis [5]. Type II ECs tend to invade surrounding tissue and metastasize, with a lower 5-year survival rate [5-8]. At the molecular level, type 1 ECs often show PTEN loss and mutations in *PI3KCA* and *KRAS* [9-12], and type 2 cancers commonly exhibit mutations in *p53* [13] and HER-2 overexpression [14].

However, this model has been challenged by the findings that many tumors actually show combined or overlapping clinical, pathological and molecular features of both classification types [15,16], suggesting that a common molecular mechanism involved in both types of cancers may exist. Consistent with this, recent molecular researches have shown that dysregulation of the PI3K/AKT signaling was found in all subtypes of EC, and associated with more aggressive disease [17-19]. Therefore, effective blocking of the PI3K/AKT pathway may be therapeutically valuable in the treatment of EC.

The epithelial-mesenchymal transition (EMT) program plays important roles in promoting tumor cell invasion, chemoresistance and cancer stem cell (CSC) properties [20,21]. Accumulating genetic and cancer biology evidence demonstrate that PI3K/AKT pathway is a central mechanism controlling EMT/CSC features, despite its definite effects on cancer cell proliferation and survival [22-25]. For example, activation of PI3K/AKT pathway was detected in radioresistant prostate cancer cells with enhanced EMT/CSC phenotypes, and the combination of PI3K inhibitor with radiotherapy induced more apoptosis in radioresistant cells, along with decreased expression of EMT/CSC markers and PI3K/AKT signaling proteins [26]. Squamous cell carcinoma lines expressing an active form of AKT produce a transcription factor Snail, which is known to promote EMT via the repression of *E-cadherin* gene [27].

MicroRNAs (miRNAs) are small non-coding RNA molecules that post-transcriptionally control the translation and stability of mRNAs. Individual miRNA can concurrently bind to multiple mRNAs and affect their expression [28]. Loss of tumor suppressive miRNAs and/or gain of oncogenic miRNAs lead to tumorigenesis and progression. In the last decade or so, miRNAs have emerged as key regulators of a wide range of genes and signals involved in modulating EMT/CSC properties, such as the PI3K/AKT pathway [29,30].

In this review, we highlight the recent advances unraveling novel roles of miRNAs in the regulation EMT/CSC phenotypes of tumor cells through targeting PI3K/AKT pathway, focusing on the potential impact of miRNAs on EMT/CSC characteristics of EC cells via targeting this pathway.

### Activation of PI3K/AKT signaling promotes EMT and CSC in EC

**Figure 1:**
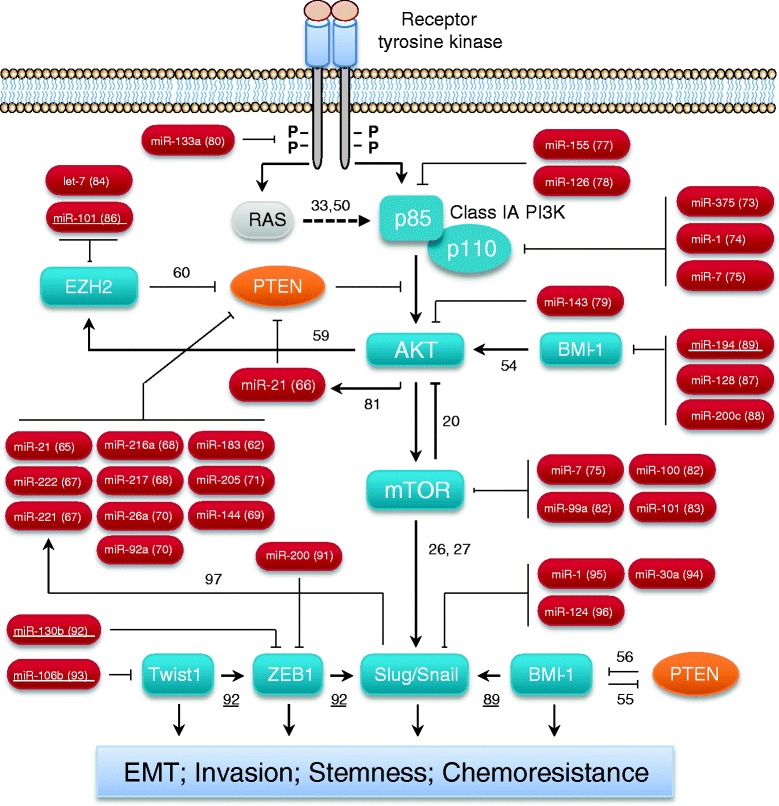
**Regulatory networks of miRNAs and PI3K/AKT pathway in controlling EMT and cancer stemness in human tumors including endometrial cancer.** Activation of PI3K/AKT signaling plays an essential role in promoting EMT and cancer stem cell phenotypes. Interactions between PI3K/AKT and other pathways regulate EMT/CSC. MiRNAs function as both upstream mediators and downstream effectors to affect PI3K/AKT pathway activities. References showing the regulatory interactions are indicated. Verified interactions in endometrial cancer were underlined.

Among three classes of PI3Ks, only classes IA PI3Ks are found to be involved in human cancers [31]. PI3K is a dimeric enzyme and consist of regulatory p85 and catalytic subunit p110 subunits [32]. Numerous important mechanisms for PI3K/AKT activation include activated receptor tyrosine kinase (RTK), RTK-induced *KRAS* activation, and genetic abnormalities in specific component of the pathway, such as loss of PTEN tumor suppressor (through deletion, gene methylation and protein stability) and *PI3KCA* (p110α) mutation or gene amplification [33,34]. The AKT kinase family has three highly homologous isoforms: AKT1, AKT2 and AKT3 [35]. Studies in breast cancer and EC cells have identified contradictory effects of AKT1 and AKT2 on cancer cell motility [36-38]. The mammalian target of rapamycin (mTOR) is a critical regulator that controls cell growth, proliferation, migration and invasion through two complexes, mTORC1 and mTORC2 [39,40]. Although activated PI3K/AKT pathway promotes mTORC1 activation, mTORC1 hyperactivation also leads to feedback inhibition of the PI3K/AKT signaling [20] (Figure 1).

In addition to affecting cell proliferation and survival, recent data suggest that dysregulation of PI3K/AKT pathway can upregulate the expression of known EMT inducers such as EZH2, BMI-1, Snail and Slug, ultimately promoting EMT and CSC features [41,42]. In immortalized mouse embryonic fibroblasts, PTEN loss results in elevated the levels of both EZH2 and BMI-1 [41]. AKT/Snail signaling activation is mechanistically associated with the acquisition of EMT/CSC properties in cisplatin-resistant lung cancer cells [42].

It has been become apparent that attenuated PTEN expression leads to constitutive activation of the PI3K/AKT pathway, which consequently contributes to tumorigenesis and metastasis of EC. Homozygous deletion of *PTEN* in *PTEN*^+/−^ mice leads to rapid formation of EC [43]. Consistent with this, conditional ablation of *PTEN* in mouse uterus is sufficient to activate the PI3K/AKT pathway and accelerate cancer cell invasiveness [44]. Furthermore, diminished AKT1 activity dramatically inhibits endometrial tumorigenesis caused by PTEN deficiency [45]. These reports indicate that hyperactivation of PI3K/AKT due to PTEN loss is a key mechanism driving initiation and invasive growth of EC.

Loss of PTEN expression is more frequently associated with metastatic malignancy [46] and worse prognosis of patients with EC [47]. When *PTEN* gene is stably transfected into *PTEN*-mutated human EC cells, cell proliferation is markedly inhibited, accompanied by a decreased level of phosphorylated-AKT (p-AKT) expression [48]. Decreased p-AKT and increased apoptosis are detected in EC cells with mutated *PTEN* in the presence of PI3K inhibitor Wortmannin [49]. Collectively, these *in vivo* and *in vitro* evidence suggests that the role of PTEN loss-of-function in facilitating PI3K/AKT-dependent endometrial carcinogenesis and progression.

Moreover, in those ECs where PTEN protein is retained, mutations in *PI3K* pathway members, such as *PIK3CA*, *PIK3R1* (p85α) or *PIK3R2* (p85β), can functionally mimic the PTEN loss, resulting in marked increases in p-AKT levels [50]. Although *KRAS* mutations are common in endometrioid ECs, it seems that *KRAS* activates independent events from PI3K/AKT pathway aberrations [50]. Epidermal growth factor receptor (EGFR) acts upstream of PI3K/AKT signal [51] and could play an initiating role to stimulate EMT in EC cells via upregulating Snail protein expression [52]. Transient transfection of constitutively active form of AKT into uterine carcinosarcoma cells results in the transactivation of *Slug*, which in turns downregulates E-cadherin expression [53].

Taken together, these studies suggest that activation of the PI3K/AKT pathway (possibly through PTEN loss, *PI3K* mutations and EGFR activation) can trigger the invasive, EMT phenotypes of EC cells through modulating the expression of EMT inducer genes.

### Interactions between PI3K/AKT and other signaling molecules regulate EMT/CSC

Oncogenes such as *BMI-1* and *EZH2* seem to act as both upstream regulators and downstream targets of PI3K/AKT pathway. The interactions between these EMT-inducing factors and PI3K/AKT pathway have been studied, and are required for the induction of EMT in tumor cells.

Overexpression of BMI-1 promotes resistance to cisplatin by increasing PI3K/AKT activity in osteosarcoma cells [54]. Likewise, another study of human nasopharyngeal epithelial cells suggest that BMI-1 transcriptionally downregulates the expression of PTEN and induces EMT through direct association with the *PTEN* locus [55]. However, in prostate cancer cells, PTEN reduces the function of BMI-1 to prevent tumorigenesis, which can be attributed to its interaction with BMI-1 in the nucleus [56], indicating that a PTEN/BMI-1 double-negative feedback loop may occur and govern EMT/CSC in certain types of cancer.

EZH2 has been reported to be required for CSC maintenance [57], and its overexpression often correlates with advanced stages and poor prognosis in diverse cancer types [58]. EZH2 has been identified as a downstream effector of PI3K/AKT pathway, and its depletion inhibits invasion and EMT in metastatic colon cancer cells [59]. However, in colon cancer stem cells the treatment with EZH2 inhibitor DZNep actually increased PTEN expression, decreased p-AKT expression and induced cell apoptosis [60]. These data suggest that the upregulation of EZH2 as a consequence of PI3K/AKT activation might increase PI3K/AKT activity by downregulating PTEN levels in CSC-like cell populations.

These studies demonstrate that feedback loops or cross-talks between PI3K/AKT pathway and other EMT/CSC-associated signaling are complex, and appear to be highly context-dependent. More studies are needed to characterize the relationship between PI3K/AKT and other important pathways in controlling EMT/CSC properties of EC cells (Figure 1).

### MiRNAs control EMT/CSC by targeting PI3K/AKT signaling in EC

As miRNAs can bind to their mRNA targets with perfect or imperfect complementary, one miRNA may concurrently influence multiple target genes in the same pathway or different cellular signaling pathways [61]. Therefore, delivery of tumor suppressor miRNAs and/or silencing oncogenic miRNAs could be a promising way to rectify aberrations in the responsible signaling pathways related to EC.

Microarray-based miRNA profiling has been successfully performed to indentify a miRNA profile distinct from normal endometrium [62]. Dysregulated miRNA levels correlate with patient prognoses in EC [63]. Of importance, miRNAs have been reported to target multiple key components in the PI3K/AKT pathway in human tumors [39,64] (Figure 1), as elaborated below.

#### PTEN

PTEN has been shown to be a direct target of miR-21 in human liver cancer [65]. Inhibition of miR-21 increases the expression of PTEN tumor suppressor, and decreases liver cancer cell proliferation, migration, and invasion [65]. Incidentally, miR-21 is also overexpressed in EC tissues, and downregulates PTEN expression via binding to the 3′-untranslated region (UTR) of *PTEN* mRNA, leading to promoted cell proliferation of EC cells [66]. In addition, miR-221 and miR-222, by targeting PTEN and raising p-AKT expression, enhances cellular migration and tumorigenicity of lung and liver cancer cells [67]. MiR-261a/217 induces EMT and increases the stem-like cell population and metastatic ability of liver cancer cells by targeting PTEN [68]. MiR-144 was reported to increase cell proliferation, migration and invasion in nasopharyngeal carcinoma through repression of PTEN [69]. Interestingly, miR-26a and miR-92a can promote cell proliferation of prostate cancer by regulating PTEN and its downstream PI3K/AKT signals [70]. MiR-205 interacts with *PTEN* mRNA and downregulates its expression in nasopharyngeal carcinoma cells [71]. MiR-205 expression is increased in ECs, and associated with decreased expression of PTEN and poorer patient overall survival [72]. This data implies a possible binding of miR-205 to *PTEN* 3′-UTR in EC cells. In another experiment, transfection of EC cells with miR-183 (a miRNA predicted to target *PTEN*) decreases PTEN protein expression [62]. However it is unclear whether miR-183 could directly suppress the 3′-UTR of *PTEN* in EC cells.

#### PI3K

*In vivo* and *in vitro* evidences support the tumor suppressor role for miR-375 in colorectal cancers [73]. MiR-375 levels are down-regulated in colorectal cancer cell lines and tissues [73]. The reporter assay confirms that miR-375 inhibits colorectal cancer cell growth through the downregulation of PIK3CA expression [73]. MiR-1, which is downregulated in lung cancer cell, suppresses cancer cell proliferation, migration, and invasion by targeting PI3KCA and decreasing p-AKT levels [74]. Furthermore, overexpression of miR-7 inhibits liver cancer cell growth and metastasis *in vitro* and *in vivo* by suppressing *PI3KCD* (p110δ) 3′-UTR [75]. *PIK3R1* is mutated in 43% of endometrioid ECs and 12% of non-endometrioid ECs [76]. Expression of mutant PI3KR1 protein leads to constitutive activation of PI3K/AKT signal [76]. Study in diffuse large B-cell lymphoma cells suggests that PI3KR1 is a direct target of miR-155 [77]. MiR-126 expression is decreased in colon cancer cells when compared to normal human colon epithelia. Forced overexpression of miR-126 suppresses tumor cell growth by repressing PI3KR2 [78].

#### AKT

AKT is a direct target of miR-143 that mediates its growth inhibition effects in bladder cancer cells [79]. Additionally, miR-133a serves as a negative regulator of breast cancer cell proliferation through targeting EGFR, thereby indirectly suppresses the levels of p-AKT [80]. Some miRNAs can function as downstream effectors of AKT, as evidenced by the rapid downregulation of miR-21 following the inhibition of the AKT pathway in colon and breast cancer cells [81]. These findings indicate that miR-21 suppresses PTEN that decreases AKT activity, resulting in the up-regulation of miR-21. Thus, miR-21 might modulate AKT expression by forming a double-negative feedback loop involving tumor suppressor PTEN [81].

#### mTOR

Several miRNAs were shown to regulate mTOR in tumor cells, including miR-7 [75], miR-99a, miR-100 and miR-101. In childhood adrenocortical tumors, miR-99a and miR-100 directly target *mTOR* 3′-UTR, and the inhibition of mTOR signaling by Everolimus greatly attenuates tumor cell growth *in vitro* and *in vivo* [82]. Furthermore, introduction of miR-101 reduces anaplastic large-cell lymphoma cell proliferation though direct repressing mTOR [83].

#### EMT inducers

Numerous miRNAs act as negative regulators of EZH2, such as let-7 family members [84] and miR-101 [85]. Let-7 family members including let-7a, −7b, −7c and -7d could strongly inhibit *EZH2* 3′-UTR luciferase activity, and repress clonogenic ability and sphere-forming capacity [84]. Restoration of miR-101 expression prevents the migration, invasion and proliferation of prostate cancer cells through the inhibition of EZH2 [85]. In aggressive EC cells, miR-101 has been found to inhibit EMT and CSC characteristics via a direct suppression of EZH2 [86]. BMI-1 is a direct target of miR-128 [87], miR-200c [88] and miR-194 [89]. These miRNAs block tumor cell self-renewal, drug resistance and metastasis. Expression levels of miR-194 are downregulated in ECs and associated with favorable survival [90]. Enforced expression of miR-194 inhibits EMT phenotype and EC cell invasion by targeting *BMI-1* [89]. MiR-200 family members reverse the EMT features of breast cancer cells through repressing ZEB1 [91]. In EC cells, we previously identified 23 miRNAs that were downregulated by mutant p53 [92]. Among them, miR-130b, which is decreased in ECs relative to adjacent normal tissues, is capable of targeting the key EMT promoter gene *ZEB1* and reverting mutant p53-induced EMT/CSC features of EC cells [92]. Another mutant p53-reponsive miR-106b can suppress EMT and cell invasion by modulating Twist1 in aggressive EC cells [93]. MiR-30a is downregulated in lung cancer and inhibits EMT by targeting *Snail* [94]. MiR-1 [95] or miR-124 [96] has been found to inhibit tumor cell invasion and EMT via regulation of Slug, which transactivates the promoter of oncogene *miR-221* in breast cancer cells [97].

Collectively, dysregulation of miRNAs contributes to altered expression of multiple genes within the PI3K/AKT pathway and are implicated in acquisition of invasive, EMT and CSC phenotype of tumor cells, supported by direct or indirect interactions between miRNAs and their target genes. However, studies evaluating the regulation of PI3K/AKT signal by miRNAs and therapeutic impact of miRNAs in EC are still rare.

### Combining miRNAs with PI3K/AKT inhibitors

Members of PI3K/AKT signaling (PTEN and mTOR) are either feedback-regulated or cross-talks to other signaling cascades [98]. Therefore, drugs targeting the PI3K/AKT pathway may more effectively treat tumors when used in combination with other targeted therapies, such as MEK inhibitors [99]. As certain miRNAs exhibit clear anti-tumor effects, one might expect the combining use of miRNAs and PI3K/AKT pathway inhibitors could enhance treatment efficacy. For example, overexpression of miR-100 coordinately represses the protein levels of mTOR and its upstream regulator FGFR3, and enhances the inhibitory effect of Everolimus on ovarian cancer cell viability [100]. In addition, a recent study suggests that the treatment of lymphoblastoid cells with mTOR inhibitors (Rapamycin and Everolimus) upregulates miR-10a expression, which in turn desensitizes cells to mTOR inhibitors response [101], indicating that miR-10a knockdown might improve the therapeutic effects of mTOR inhibitors. Thus, the combined use of PI3K/AKT inhibitors and miRNAs would be an attractive and possible therapeutic option.

### EMT and tumor growth

A reversion of EMT, or MET is believed to allow tumor cells to restart their proliferation in the metastatic site [102]. Snail-induced EMT attenuates the cell cycle [103], and turning off Twist1 was found to increase proliferation in an *in vivo* skin tumor model [104]. However, Snail expression correlates with E-cadherin downregulation, actually increases proliferation in the hair bud where skin cells maintain the epithelial phenotype [105], thus the profound remodeling of the cytoskeleton (complete EMT) could be linked to decreased cell proliferation [106], whereas incomplete or transient activation of the EMT program might conversely induce cell proliferation. Consistent with this notion, complete and incomplete EMT phenotypes have been identified in human cancers, and associated with worse and relatively better survival rates, respectively [107,108]. Interestingly, EC cells undergo EMT tend to exhibit enhanced proliferation, invasiveness and cell scattering (rather than a complete conversion to a mesenchymal morphology) [86,109,110], implying that a partial EMT program may often occur in EC cells. In addition, certain EMT inducer such as Twist1 cooperates with co-factors to promote tumor cell proliferation by abrogating cellular senescence [111]. These studies suggest that EMT processes, possibly induced by PI3K/AKT pathway, may confer increased EC cell invasion without inhibiting cell proliferation.

## Conclusions

Growing evidence suggests that PI3K/AKT activation is vital to the induction of EMT and CSC properties in tumor cells. MiRNAs control cancer initiation, progression and metastasis, and function as both upstream mediators and downstream effectors to affect PI3K/AKT pathway activities. Introduction of tumor suppressive or knockdown of oncogenic miRNAs would be a promising approach to inhibiting the PI3K/AKT pathway in EC. The combination of miRNAs with PI3K/AKT inhibitors and inhibitors against other signals that cross-talk with PI3K/AKT pathway, might yield promising therapeutic effects.

Challenges of miRNA-based therapies in EC probably include the risk of unintended off-target effects [20], the necessity of eliminating CSC as well as non-CSC tumor cells [112], and the identification of alterations in the 3′-UTR of target gene, such as shortened 3′-UTR sequence and 3′-UTR mutations, which could disrupt the binding capacity of a certain miRNA [113-115]. Next-generation sequencing technologies have been used to identify some miRNAs that are expressed in a tissue-specific manner [116,117], raising the possibility that tissue-specific miRNAs might be used to specifically target tumor cells and avoid off-target effects. Our new knowledge of the roles of miRNAs and their targets in regulating PI3K/AKT pathway will expand the utility of miRNAs for suppressing EMT and CSC in EC.
